# Molecular and Structural Parallels between Gluten Pathogenic Peptides and Bacterial-Derived Proteins by Bioinformatics Analysis

**DOI:** 10.3390/ijms22179278

**Published:** 2021-08-27

**Authors:** Diego S. Vazquez, Hanna M. Schilbert, Veronica I. Dodero

**Affiliations:** 1 Grupo de Biología Estructural y Biotecnología (GBEyB-IMBICE), Departamento de Ciencia y Tecnología, Universidad Nacional de Quilmes, Roque Sáenz Peña 352, Bernal B1876BXD, Buenos Aires, Argentina; dsvazquez@conicet.gov.ar; 2 Consejo Nacional de Investigaciones Científicas y Técnicas (CONICET), Av. Rivadavia 1917, Ciudad Autónoma C1033AAJ, Buenos Aires, Argentina; 3 Department of Chemistry, Organic Chemistry OCIII, Universität Bielefeld, Universitätsstraße 25, 33615 Bielefeld, Germany; h.schilbert@uni-bielefeld.de

**Keywords:** celiac disease, non-celiac gluten sensitivity, SH3 and WW domains, 33-mer peptide, p31-43 peptide, gliadin epitopes, pathogens, sequence similarity, innate immune response

## Abstract

Gluten-related disorders (GRDs) are a group of diseases that involve the activation of the immune system triggered by the ingestion of gluten, with a worldwide prevalence of 5%. Among them, Celiac disease (CeD) is a T-cell-mediated autoimmune disease causing a plethora of symptoms from diarrhea and malabsorption to lymphoma. Even though GRDs have been intensively studied, the environmental triggers promoting the diverse reactions to gluten proteins in susceptible individuals remain elusive. It has been proposed that pathogens could act as disease-causing environmental triggers of CeD by molecular mimicry mechanisms. Additionally, it could also be possible that unrecognized molecular, structural, and physical parallels between gluten and pathogens have a relevant role. Herein, we report sequence, structural and physical similarities of the two most relevant gluten peptides, the 33-mer and p31-43 gliadin peptides, with bacterial pathogens using bioinformatics going beyond the molecular mimicry hypothesis. First, a stringent BLASTp search using the two gliadin peptides identified high sequence similarity regions within pathogen-derived proteins, e.g., extracellular proteins from *Streptococcus pneumoniae* and *Granulicatella* sp. Second, molecular dynamics calculations of an updated α-2-gliadin model revealed close spatial localization and solvent-exposure of the 33-mer and p31-43 peptide, which was compared with the pathogen-related proteins by homology models and localization predictors. We found putative functions of the identified pathogen-derived sequence by identifying T-cell epitopes and SH3/WW-binding domains. Finally, shape and size parallels between the pathogens and the superstructures of gliadin peptides gave rise to novel hypotheses about activation of innate immunity and dysbiosis. Based on our structural findings and the similarities with the bacterial pathogens, evidence emerges that these pathologically relevant gluten-derived peptides could behave as non-replicating pathogens opening new research questions in the interface of innate immunity, microbiome, and food research.

## 1. Introduction

Celiac disease (CeD) is a chronic, small-intestinal T-cell-mediated autoimmune disease triggered by the ingestion of dietary gluten from common food grains such as wheat, rye, and barley, in genetically predisposed individuals with a prevalence of about 1% in the general population with regional differences [1]. Other gluten-related disorders (GRDs) such as non-celiac gluten sensitivity (NCGS) are less understood and have a prevalence rate between 0.5% and 5% [2]. Nowadays, the only proven treatment for GRDs is strict and life-long adherence to a gluten-free diet [3].

Viral and bacterial pathogens have long been suspected of triggering immune responses that are directed toward autoimmunity in CeD. In 2002, the group of Khosla showed that the immunodominant gluten fragment has sequence similarity with pertactin, a highly immunogenic protein from the bacterium *Bordetella pertussis*; however, this result was not further investigated [4]. It recently identified and characterized a number of mimics of HLA-DQ2.5-restricted gliadin determinants derived from the commensal bacterium *Pseudomonas fluorescens*, activating disease-relevant gliadin reactive T cells isolated from CeD patients [5]. This report was a major proof of concept that a molecular mimicry mechanism may trigger CeD.

Beyond T-cell activation in CeD, it was also proposed that gluten proteins have functional similarities with non-replicative pathogens such as prions [6,7]. The main issue with gluten proteins is that the human digestive proteases can only partially degrade them leading to a mixture of peptides that elicit immune and toxic effects in predisposed individuals and cell lines [8]. The mononuclear phagocytic system (MPS), composed mainly of macrophages, neutrophils, and dendritic cells, is part of the first line of defense against pathogens. Pathogens are recognized by various immune cells, such as macrophages and dendritic cells, via pathogen-associated molecular patterns (PAMPs) on the pathogen surface, which interact with complementary pattern-recognition receptors (PRRs) on the pathogen surface the immune cell surfaces. While PRR activation is a central component to the resulting immune response, innate cells also respond to the size and shape of the pathogens and the spatial organization of PAMPs on the bacterial surface. In recent years, it has become evident that the MPS can engulf other non-self-systems such as synthetic nanoparticles generating in many cases an immune response against the foreign nanosystem [9,10]. From a pathological standpoint, there are some concerns in the possibility that the presence of nanostructures could exacerbate or prolong PRR-driven inflammatory reactions, leading to uncontrolled tissue-damaging inflammation. In this context, it was demonstrated that pepsin digests of gliadin form spontaneously amyloid-like structures that trigger genes in the gut epithelial cell model Caco-2 involved in recruiting specialized immune cells [11]. Furthermore, the most studied gluten peptides are 33-mer peptides and the p31-43 fragment that form peptide nanostructures, too. Both peptides trigger the adaptive or an innate immune response, respectively, in CeD patients, animal models, and cell lines culture [12,13,14,15,16].

The immunodominant 33-mer peptide comprises residues 57 to 89 of α-2-gliadin (LQLQPFPQPQLPYPQPQLPYPQPQLPYPQPQPF). In total, 39% of the 33-mer peptide residues are prolines leading to a type-II polyproline (PPII) conformation in solution, which is known to be bound to MHC class-II molecules [14]. When the 33-mer accumulates, it forms superstructures ranging from dimers to nano- and microstructures ranging from 10 nm to more than 1 µm [17,18,19]. Importantly, there is a concentration-dependent structural transition from PPII toward a parallel β-sheet conformation accompanied by the formation of large superstructures that activate macrophages in vitro via Toll-like receptor 4 (TLR4) and TLR2 [20,21]. The mentioned secondary structures are essential in protein-protein interaction and might function as a signaling component. The high percentage of Q makes this peptide amphiphilic favoring the formation of hydrogen bonds [22], a key factor in the self-assembling process, stressing the importance between sequence and morphology [17].

The second most studied gliadin peptide is the toxic p-31-43, a 13-mer peptide comprising the amino acids 31 to 43 (FPGQQQPFPPQQP). Although the p31-43 peptide is not presented by the HLA-DQ2 [23], the reason for its toxicity in CeD remains unknown. The mechanism by which p31-43 induces an immune response in celiac patients has been recently attributed to its effects on the endocytic compartment affecting several cell functions such as proliferation, cell motility, and innate immunity activation [23]. Nanayakkara et al. [24] recently proposed that p31-43 induces the IFN-α-mediated innate immune response in the CaCo-2 enterocyte cell-line by activating the TLR7 signaling pathway mimicking the immune response triggered by viruses. Recently, it was reported that p31-43 has a PPII secondary structure and can, as well as 33-mer, self-assemble under physiologically relevant conditions [25]. Even more, p31-43 oligomers have been proposed to be responsible for activating the inflammasome in murine models [26].

Finally, it is reported that gliadin acts as a modulator of human microbiota [27], and it is not clear if changes in the microbiota lead to gluten-related disorders or that the presence of gluten peptides in the gut leads to changes in the microbiota [28,29].

Based on the reported experimental evidence in cell lines, animal models, and patients’ biopsies, it may also be possible that gluten fragments share additional structural and morphological similarities with bacterial or viral pathogens beyond the established T-cell cross-reactivity in CeD, which would be particularly relevant in the understanding of the innate immune activation.

**Figure 1:**
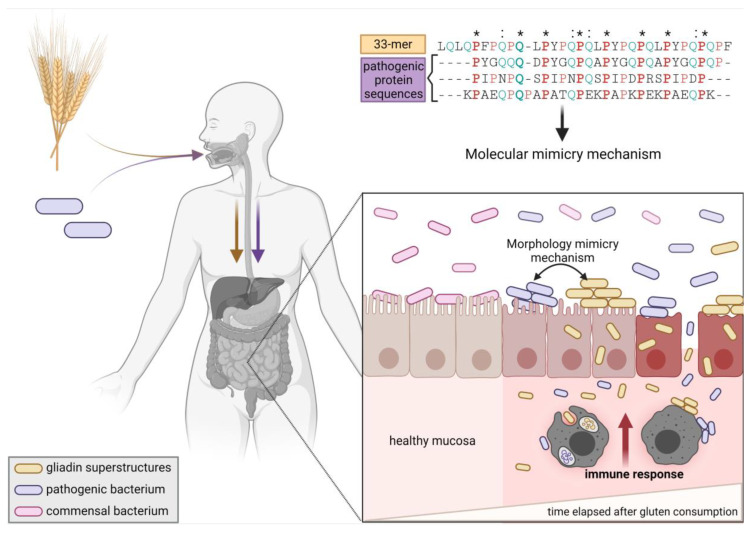
Cartoon summarizing the results and hypothesis presented here. Gluten peptides and pathogens can both enter the intestinal lumen through oral intake. Based on this work, gluten peptides share high sequence, structural and morphological similarities with different proteins found in bacterial pathogens. Some of them share putative T-cell epitopes related to celiac disease (CeD), reinforcing the idea of molecular mimicry as the trigger of CeD (conserved amino acids in the 33-mer sequence and the bacterial proteins are shown (*)). On the other side, a new concept of morphology mimicry is presented, where the immune cells recognize gluten superstructures as bacterial pathogens because of their similar morphology, starting an innate immune response. Exposure to most pathogens and gluten is necessary but not sufficient to cause disease because the genetic background and susceptibility, and dose effects, are additional determinants for disease progression (Created with biorender.com, accessed on 23.08.21, under academic license terms).

Herein, we showed using bioinformatics that the two most relevant gliadin peptides 33-mer and p31-43 have sequence and structural similarities with proteins found in bacterial pathogens giving novel insights into the early innate immune response. First, we performed a stringent Basic Local Alignment Search Tool protein (BLASTp) search [30] based on the 33-mer and p31-43 sequences outside the *Gramineae* family and identified high sequence similarity with proteins from different bacterial pathogens. Among them are *Streptococcus pneumoniae* and *Granulicatella* sp., two well-known causative agents of diverse human diseases. Second, we investigated the spatial localization of both gluten fragments in α-2-gliadin using molecular modeling and calculated sequence-based intrinsic disorder (ID) tendency and subsequent calculations of solvent-accessible surface area (SASA) and aggregation propensity. Next, we employed tridimensional homology modeling to analyze the structural similarities between the target pathogen-derived proteins and the 33-mer and p31-43 sequences in α-2-gliadin, performing a comprehensive literature search to identify pathogenic similarities. Importantly, in those pathogenic proteins, with sequence similarity with the 33-mer fragment, putative T-cell epitopes, and SH3-binding domains were found. Finally, the shape and size similarities between the superstructures of the gliadin peptides and the reported pathogens were presented and connected with morphology mimicry and dysbiosis (Figure 1).

## 2. Results and Discussion

### 2.1. High Sequence and Structural Similarity of 33-mer and p31-43 Sequences in Pathogen-Derived Proteins

One key unsolved question in GRDs is the environmental triggers that promote the diverse reactions to gluten proteins observed in susceptible individuals. Thus the factors involved in the switch from tolerance to disease need to be investigated. Initially, we performed an in-depth sequence and structural analysis of foreign proteins sharing high sequence similarity regions with the 33-mer and p31-43 sequences. The top-five BLASTp hits using the 33-mer and the p31-43 sequence as queries are presented in Table 1. Overall the identified similarity regions reached up to 68% sequence identity in the 33-mer and up to 85% for the p31-43 similarity regions. Importantly, 5 out of 10 identified proteins belonging to the host organisms known to be pathogenic for humans to different degrees. The complete information obtained in this work is summarized in Table 1 and discussed in the following sections.

### 2.2. The Structural Evaluation of Gliadin Peptides Harboring Pathogen-Related Proteins Similarity Regions and Their Function

#### 2.2.1. Spatial Localization and Structural Information of the Relevant Gliadin-Derived Peptides in the α-2-Gliadin 3D Model

**Figure 2:**
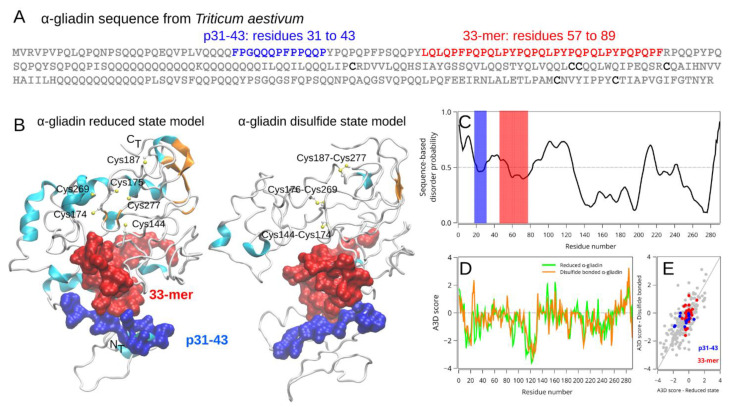
Structural characterization of the reduced and oxidized states of α-2-gliadin of *Triticum aestivum*. Primary sequence of wheat α-2-gliadin where the p31-43 (blue) and 33-mer (red) regions are highlighted (**A**). Tridimensional structure of α-2-gliadin in the obtained previously [19] and the new disulfide state model (**B**). The immunogenic regions are in *surface* representation using the same color reference as in panels A and C, and the cysteine pairs involved in disulfide bonds are marked for clarity. Sequence disorder prediction was calculated using the PrDOS server (**C**), and the aggregation propensity was calculated using AGGRESCAN 3D (**D**) for both oxidation states. The horizontal gray line in panel C marks the 5% threshold (false positive rate), while aggregation-prone regions have positive values, respectively. The *per* residue correlation between both oxidation states is shown in panel (**E**).

Wheat gluten proteins can be classified into soluble gliadins and insoluble low and high-molecular-weight glutenins depending on their solubility in aqueous/alcohol mixtures [37]. Moreover, gliadins can be classified into α/β-, γ- and ω-gliadins according to their primary sequence and electrophoretic mobility [37]. Gliadins are named prolamins because of their high content of proline (Pro) and glutamine (Gln) amino acids, having self-assembly capabilities [20,38]. More than 50 immunogenic epitopes are present in different classes of wheat gluten proteins [13]. Considering the absence of structural information about gliadins because of their intrinsic disorder (ID) (Figure 2), we previously built a tridimensional model of α-2-gliadin [19]. Here, we present an improved model (Figure 2B) that incorporates the disulfide bridges between Cys144-Cys177, Cys175-Cys269, and Cys187-Cys277 [39]. Interestingly, the presence of the disulfide bonds leads to an increase in conformational disorder (Figure 2B). However, despite the notorious topological change, the structure-based aggregation profile remains nearly unchanged (Figure 2D,E). Interestingly, both proteolytic-resistant peptides are predicted to be exposed to the solvent and are located in disordered regions that are spatially closed in both tridimensional models (Figure 2B,C).

#### 2.2.2. The Homology Models of Pathogen-Related Proteins Containing Sequences Similar to 33-mer Gliadin Peptide and Their Function

**Figure 3:**
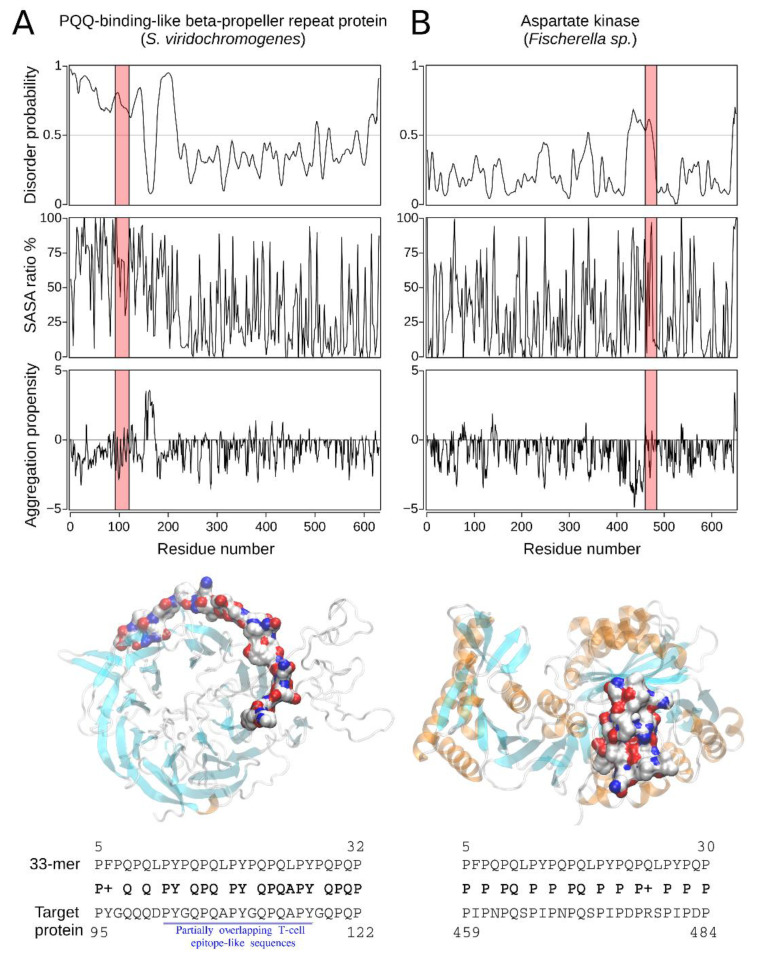
Sequence and structural characterization of target proteins found by BLASTp search of the 33-mer peptide. For both target proteins from *Streptomyces viridochromogenes* (**A**) and *Fischerella* sp (**B**) the region sharing high sequence similarity with the 33-mer sequence is shown in a red box. The disorder prediction (top panel) of the best hits was calculated by the PrDOS server setting a 5% threshold (false positive rate, gray horizontal line). The percentage of solvent-accessible surface area (SASA) was calculated after an energy minimization of the tridimensional models (middle panel). The structure-based aggregation propensity (lower panel) was calculated using AGGRESCAN 3D, where positive values indicate an aggregation-prone region (horizontal line). The tridimensional structure of the target proteins was modeled using the PHYRE2 server. The regions sharing high sequence similarity with the 33-mer sequence are shown in *surface* representation. In addition, the sequence alignments between the 33-mer sequence and the high similarity region of the target proteins are shown at the bottom. Partially overlapping copies of T-cell epitope-like sequences are blue underlined in the alignment of panel (**A**).

The PQQ-repeat protein of *S. viridochromogenes* harbors one transmembrane domain of pyrroloquinoline quinone (PQQ) repeat sequence (Table 1 (A): Hit#1). This suggests that it belongs to the quinoprotein alcohol dehydrogenase-like protein superfamily, which has a β-propeller-like fold (Figure 3A). This architecture is composed of four-stranded antiparallel and twisted β-sheets, which are radially distributed, forming a central tunnel similar to a TIM-barrel fold (Figure 3A). The first ~150 amino acids could not be partially modeled due to the high sequence disorder (Figure 3A). In general, the PQQ serves as a redox cofactor for several enzymes, e.g., bacterial dehydrogenases [40]. A study showed that dietary PQQ exposure resulted in apparent antioxidant potential changes and significant decreases in the levels of plasma C-reactive protein, IL-6, urinary methylated amines such as trimethylamine N-oxide, and changes in urinary metabolites consistent with enhanced mitochondrial-related functions [41].

The aspartate kinase from *Fischerella* (Table 1 (A): Hit#2; Figure 3B) contains a putative nucleotide and Mg^2+^ ion-binding site, as well as two putative allosteric regulatory sites. Aspartate kinases catalyze the ATP-dependent reaction of L-aspartate to 4-phospho-L-aspartate and ADP and are involved in amino acid metabolism [32].

The 2036 residues-long hypothetical protein of unknown function from *Granulicatella* sp. contains a F/YSIRK-type signal peptide (Table 1 (A): Hit#3) with the sequence FSIRKxxxGxxS [42], which suggests a transmembrane destination in line with our results presented below. Due to the lack of structural data of related proteins, the intrinsic disorder profile (Figure S1), and the large size of the protein, we could not model this protein to obtain 3D structural information. However, the C-terminal LPxTG motif strongly suggests that this protein can be processed by sortases [43]. Sortases are cysteine transpeptidases, which decorate the protein surfaces and are extensively found in Gram-positive bacteria [43]. Proteins modified by sortases are often involved in infection, virulence, and colonization processes [44,45].

**Figure 4:**
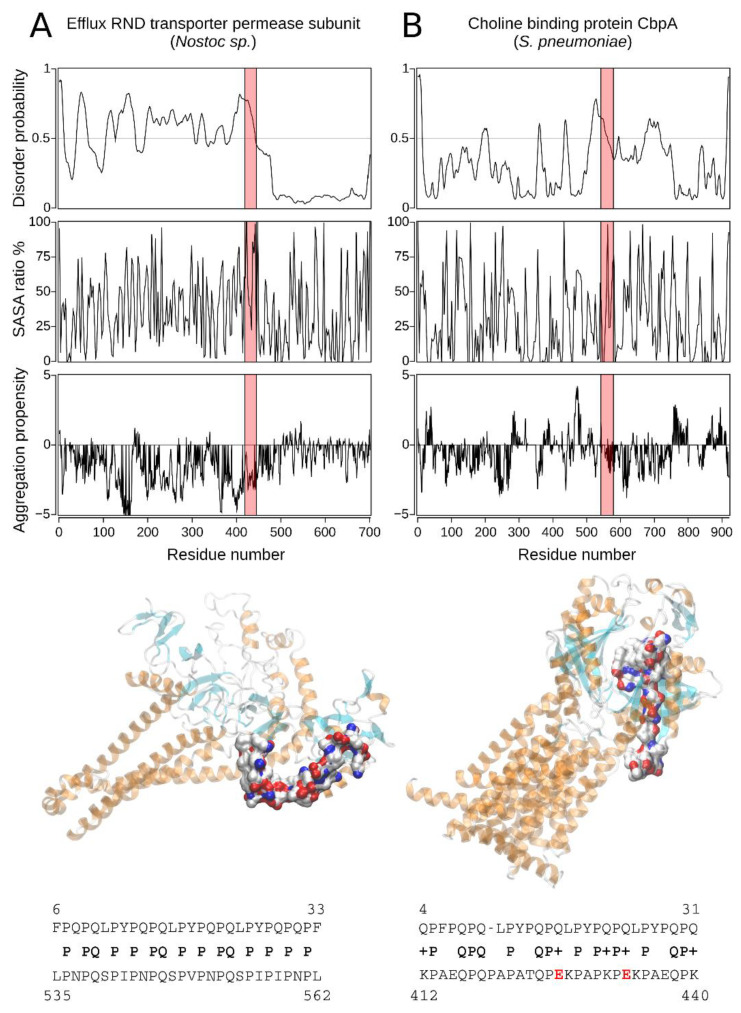
Sequence and structural characterization of target proteins found by BLASTp search of the 33-mer peptide. For both target proteins from *Nostoc* sp. (**A**) and *Streptococcus pneumoniae* R6 (**B**) the region sharing high sequence similarity with the 33-mer sequence is shown in a red box. The disorder prediction (top panel) of the best hits was calculated by the PrDOS server setting a 5% threshold (false positive rate, gray horizontal line). The percentage of solvent-accessible surface area (SASA) was calculated after an energy minimization of the tridimensional models (middle panel). The structure-based aggregation propensity (lower panel) was calculated using AGGRESCAN 3D, where positive values indicate an aggregation-prone region (horizontal line). The tridimensional structure of the target proteins was modeled using the PHYRE2 server. The regions sharing high sequence similarity with the 33-mer sequence are shown in *surface* representation. In addition, the sequence alignments between the 33-mer sequence and the high similarity region of the target proteins are shown at the bottom. Relevant amino acid exchanges, such as Gln to Glu, are marked in red in the alignment of panel (**B**).

Choline-binding proteins (cbps) such as cbp A from *S. pneumoniae* (Table 1 (A): Hit#4, Figure 4B) are non-covalently bound cell-surface proteins involved in virulence [46,47]. Cbp A increases the expression of intercellular adhesion molecule 1 (ICAM-1, also known as CD54), an early inflammatory marker [47]. Therefore, cbp A induces the transcription and release of proinflammatory molecules by human alveolar epithelial cells [47]. ICAM-1 is necessary for the initial contact of circulating leukocytes with activated endothelium and is up-regulated or constitutively expressed in response to proinflammatory cytokines on endothelial cells, activated lymphocytes, monocytes, and epithelial cells [48]. Further, the histological evaluation revealed increased expression of ICAM-1 in most cells of the lamina propria in jejunal biopsies from CeD patients.

Additionally, it was observed that ingestion of gluten induced a rapid increase in ICAM-1 expression in CeD patients compared to healthy control and patients with non-celiac gluten sensitivity [49]. Importantly, a specific variant of ICAM-1 with an arginine at position 241 is known to be a predisposing factor for the development of CeD in adulthood [50]. Cbp A has also been shown to bind the secretory component of immunoglobulin A, the complement protein C3 [51], and recently facilitated invasion into and through nasopharyngeal epithelial cells by interacting with the human polymeric immunoglobulin receptor [52]. This receptor is expressed by mucosal epithelial cells binding polymeric immunoglobulins and releasing them as secretory antibodies in the mucosa [53]. In this direction, it was reported that intestinal transport of intact 33-mer and p31-49 was blocked by polymeric and secretory IgA (SIgA) and by soluble CD71 receptors, pointing to a role of SIgA-gliadin complexes in this abnormal intestinal transport [54].

The Hit#5 from *Nostoc* sp. PCC 7524 is predicted to be a cation/multidrug RND (Resistance-Nodulation-Division) efflux pump with transporter activity and belongs to the AcrB/D/F protein family (Table 1 (A): Hit#5, Figure 4A). AcrB/D/F proteins are integral membrane proteins [55]. Some are involved in multidrug resistance [55]. For example, AcrB cooperates with the membrane fusion protein AcrA and an outer membrane channel TolC and forms homotrimers [55].

#### 2.2.3. The Homology Models of Pathogen-Related Proteins Containing the p31-43 Similar Sequence and Their Function

**Figure 5:**
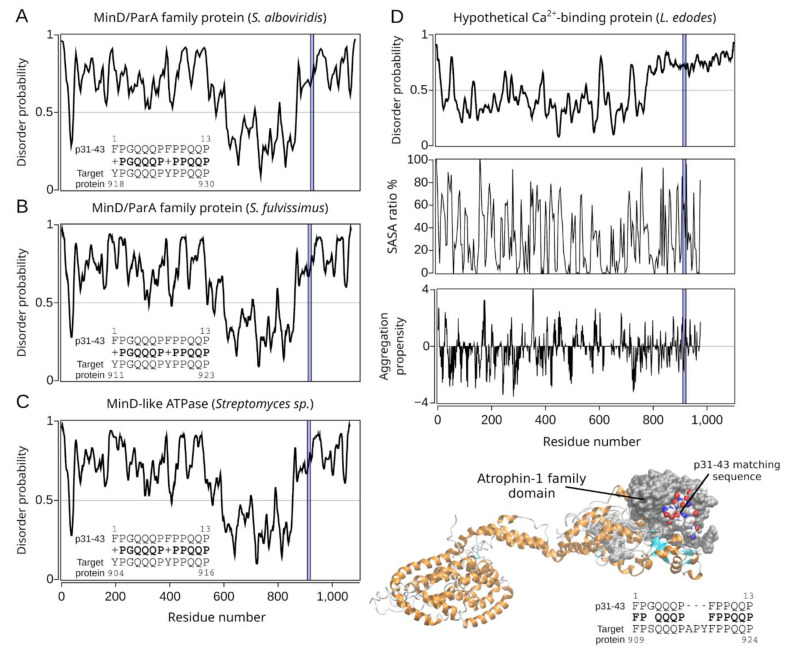
Sequence and structural characterization of target proteins found by BLASTp search of the p31-43 peptide. In all cases, the regions sharing high sequence similarity with the p31-43 sequence are shown in a blue box. The sequence-based disorder prediction for the MinD/ParA proteins from *Streptomyces alboviridis* and *Streptomyces fulvissimus*, the MinD-like ATPase from *Streptomyces* sp. ScaeMP-e48, and the hypothetical calcium-binding protein from *Lentinula edodes*, are shown in panels (**A**–**D**), respectively, setting a 5% threshold (false positive rate, gray horizontal line). For the latter, the percentage of solvent-accessible surface area (SASA) *per* residue and aggregation propensity are shown alongside the target model at the bottom right. The atrophin-1 domain is represented in surface (gray), and the region with high sequence similarity to the p31-43 sequence of α-2-gliadin is represented in van der Waals. In addition, the sequence alignment with the target protein is shown.

Three MinD/ParA family proteins from *Streptomyces sp*. were identified (Table 1 (B): Hits#1–3). With an overall similar ID profile for all three proteins and high content of sequence disorder, their tridimensional structure could not be modeled (Figure 5A–C). The identified MinD/ParA family proteins contain an FlhG domain characterized through a conserved nucleotide phosphate-binding motif and involved in diverse cellular functions such as chromosome partitioning or flagellar assembly [56]. ParA and MinD are members of a larger family of ATPases, defined by the specific variant of their Walker A sequences (KGGXXK[ST]) [57]. In general, Par is a transportation system for DNA [58]. Indeed, the partition ATPase (ParA) provides energy for DNA transportation and is related to other ATPases that localize components within a bacterial cell [58].

Regarding its function as a DNA-transport system, the protein possesses non-specific DNA binding activity. It binds DNA with a patch of basic amino acids located at the C-terminus [59,60,61]. However, the membrane-associated ATPase MinD is required for localizing the *Escherichia coli* cell division machinery at mid-cell by acting as cell division inhibitor and activating MinC [62]. Interaction of MinD with MinC was predicted to be located at the residues around the nucleotide-binding site in MinD [63]. The positioning of MinD/ParA proteins is either due to self-organization on a surface or reliance on a landmark protein that functions as a molecular beacon [64].

Finally, the hypothetical calcium-binding protein from *L. edodes* (Table 1 (B): Hit#4) shows a low ID score except for a large disordered C-terminal region (Figure 5D). Thus, the hypothetical protein was modeled with high accuracy for more than 95% of the amino acids (Figure 5D).

### 2.3. Primary Structure Analysis and Potential Functions

#### 2.3.1. CeD-T-Cell Epitopes

The molecular mimicry hypothesis considers that pathogens and/or pathogen-derived proteins/peptides sharing molecular and structural similarities with gluten fragments trigger a strong humoral immunological response. After the primary disease is treated, the host immune system recognizes gluten pathogenic fragments as if they are signatures of the bacterial pathogens [65,66].

Molecular mimicry is nowadays recognized as a pathogenic mechanism by which infectious or chemical agents may induce autoimmunity and occurs when foreign and self-peptides share similarities at the sequence or structural level, favoring the activation of autoreactive T or B cells in predisposed individuals [67]. There is extensive documentation of autoimmune diseases that have been associated by molecular mimicry with foreign pathogens such as acute gastroenteritis by rotavirus [68], autoimmune thyroid diseases by *Helicobacter pylori* and Hepatitis C virus [69], systemic lupus erythematosus by *Leishmania* sp. [70], acute rheumatic fever by group A *streptococci* [71,72] and recently the SARS-CoV-2 coronavirus, the cause of the worldwide COVID-19 pandemic disease, with the Guillain-Barré syndrome [73].

In this context, the 33-mer sequence is responsible for the adaptive immune response in CeD because it contains six partially overlapping copies of canonical T-cell epitopes [14]: three copies of the DQ2.5-glia-α1- (PF/YPQPQLPY) and the DQ2.5-glia-α2 (PQPQLPYPQ) epitope [74]. Thus, one of the most relevant findings is the presence of two partially overlapping copies of the PYGQPQAPY motif in the PQQ-repeat protein of *S. viridochromogenes* (Table 1 (A): Hit#1), which is similar to the DQ2.5-glia-α1 epitope (Figure 3A). The two mismatches are P to G at position 3 and L to A at position 7 (Figure 3A). Given more than 70 variants found in the canonical sequence of *Triticum* species [74] and considering the conservative physicochemical properties and size of the replacements, the identified sequence could be a potential immunodominant T-cell DQ2 epitope. Interestingly, the RND efflux pump from *Nostoc* sp. contains three partially overlapping copies of the PNPQSPXP sequence where X is I or V (Figure 5). Furthermore, there are two or three Q to E variations in two pathogens-related proteins, the Efflux RND transporter permease and F/YSIRK-type protein (Table 1 (A): Hits#5 and #3; Figure 4). The introduction of a negative charge through deamidation leads to a higher binding affinity of the 33-mer gliadin sequence to DQ2/8 MHC class-II molecules on the antigen-presenting cell membrane [75], increasing the immunogenicity of this sequence. Even more, the deamidated variant of the 33-mer peptide is known to be an extremely potent T-cell stimulator in comparison with other gluten-derived peptides [76,77]. The heterogeneity of gluten protein sequences makes it challenging to elucidate the entire repertoire of potential peptides involved in the pathogenesis of CeD [78], and these sequences need to be experimentally investigated.

#### 2.3.2. SH3/WW Domains Binders

Although the functions of the similarity regions of the pathogen proteins are unknown, their localization and polyproline II (PPII) propensity (due to the high content of proline and glutamine amino acids) suggest that they could be potential targets in protein-protein interaction with SH3 domains. Many proteins of the Src kinases family carry small modules named Src Homology 3 (SH3) domains with a characteristic β-barrel fold, which usually enables the binding to proline-rich sequences with a PPII conformation [79]. The SH3 domains are found ubiquitously in all eukaryotes, some prokaryotes, and even viruses [79]. With more than 300 SH3 domains encoded in the human genome, these are crucial elements in protein-protein interactions in several signal transduction pathways [80].

**Figure 6:**
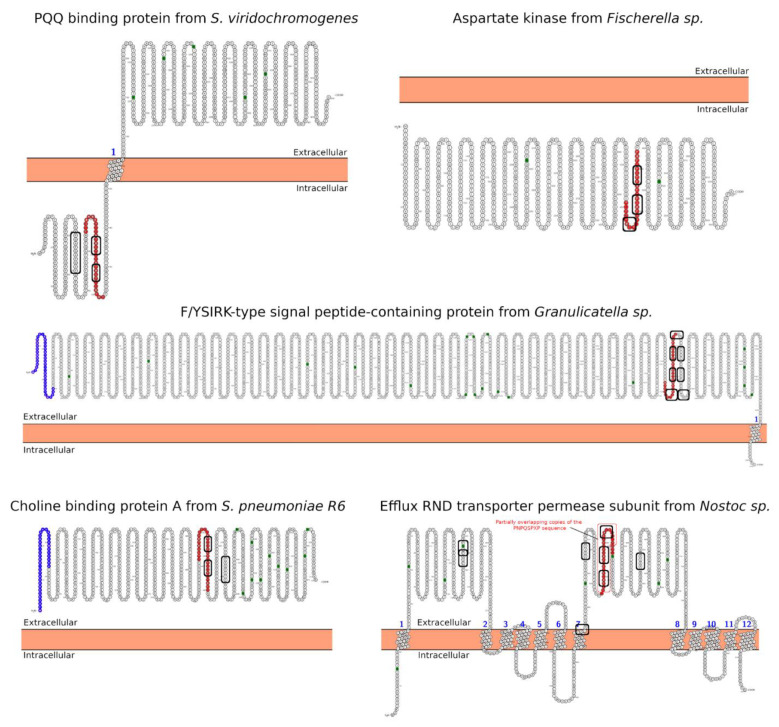
Predicted subcellular localization for the BLASTp hits proteins using the 33-mer sequence as query. The localization of the high similarity sequence regions from the five best BLASTp hits is shown in red. The SH3-binding motifs are highlighted with a rectangle. The analysis was performed using the Protter server [82]. The partially overlapping copies of the PNPQSPXP sequence in the RND efflux pump protein are shown as a red rectangle. The signal peptides are shown in blue and N-glycosylation motifs are marked as green squares.

**Figure 7:**
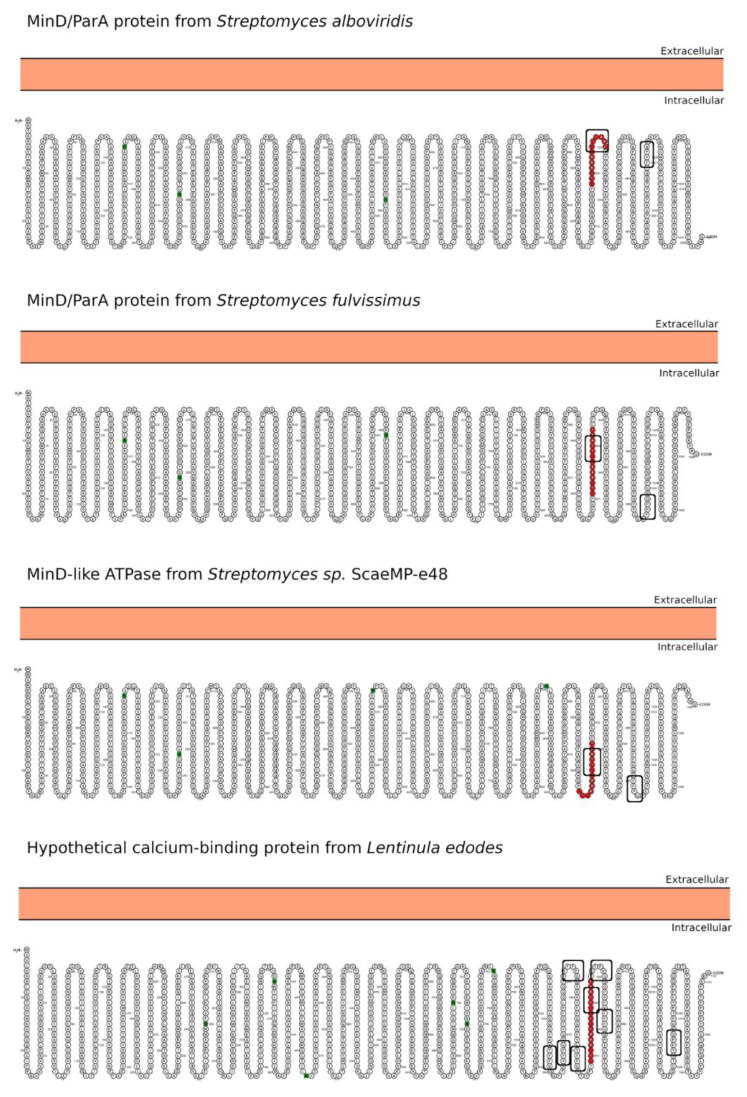
Predicted subcellular localization for the BLASTp hits proteins using the p31-43 sequence as a query. The localization of the high similarity sequence regions from the four best BLASTp hits is shown in red. The Y/FPPQ-binding motif recognized by the WW domain is highlighted with a rectangle. The analysis was performed using the Protter server [82]. The N-glycosylation motifs are marked as green squares.

In addition, the WW domain mediates specific protein-protein interactions with short proline-rich or proline-containing motifs [81]. The SH3- and WW domains usually bind to PxxP and xPPx motifs, respectively. In this regard, both the 33-mer and p31-43 peptides could be SH3-binding partners due to their PQLP and PQQP sequences, respectively. The 33-mer similarity sequences for the PxxP SH3-binding motif are found in the form of PEKP (*Granulicatella* sp.); PEKP, PAQP, and PSTP (*S. pneumoniae*); and PSSP, PINP, PFIP, PKIP, PQSP, and PKLP (*Nostoc* sp.) who are predicted by Protter web server [82] to be extracellular (Figure 5). Meanwhile, PQSP and PRSP (*Fischerella* sp.) and PQAP (*S. viridochromogenes*) would be located intracellular (Figure 6). On the other hand, only the p31-43 and its similar pathogen sequences have the Y/FPPQ sequence predicted to be in the cytoplasm and with the potential capability to bind the WW domain (Figure 7) [83,84]. Thus, both sequences found in gliadin and the pathogen-related proteins may potentially bind to a plethora of proteins in vivo, which could lead to several unknown metabolic (dis-)functions.

### 2.4. Gliadin Peptide Superstructures, Pathogen Morphology, and Dysbiosis as Triggers of the Innate Immune Response: The Hypothesis

The significance of the term pathogen as “*things*” capable of causing human diseases was historically associated with microorganisms. Nevertheless, Griffith’s proposed the pathogenic role of Prions proteins in scrapie in 1967, and the seminal work of Prusiner and coworkers in 1982 laid the groundwork to reconsidering the meaning of the term. Nowadays, the term pathogen was replaced by the widely accepted *infectious agent,* which includes biomolecules such as proteins. However, as suggested by Methot and Alizon, a more complex scenario needs to be considered as an ecological, evolutionary, and immunological context takes a prominent role in the host-pathogen interaction [85].

**Figure 8:**
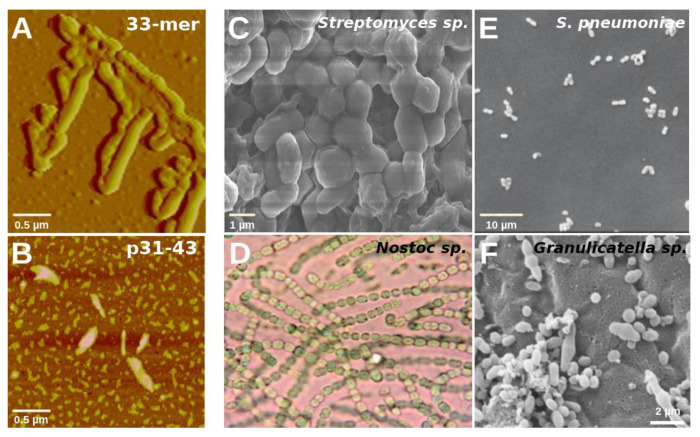
Morphology of host organisms associated with the 33-mer and p31-43 peptide BLASTp hits. Topology of the 33-mer ((**A**), adapted with permission from Reference [18]) and p31-43 oligomers ((**B**), adapted with permission from Reference [25]) alongside with the morphology of representative host organisms found in our work: *Streptomyces* sp. VN1 ((**C**), adapted from Reference [90]), *Nostoc* sp. *EA03* ((**D**), adapted from Reference [91]), *Staphylococcus pneumoniae* TIGR4 ((**E**), adapted from Reference [92]) and *Granulicatella adiacens* ((**F**), adapted from Reference [93]). Images in panels (**C**–**F**) are under CC BY licenses.

In this broad scenario, it could also happen that gluten peptides that share structural/morphological similarities with pathogens have latent pathogenicity and, although initially innocuous to the host, after their accumulation and oligomerization with the conformational transition toward amyloid structures, start to be recognized by the host innate immune as non-replicating pathogens. Interestingly, all the identified host pathogens in the BLASTp search share a rod-like morphology that is linearly organized. It is very similar to the morphology of the 33-mer superstructures and, to some extent, to p31-43 oligomers (Figure 8).

Several studies in vitro and in vivo agreed that particles in the size range of 40 nm to 10 μm are the most immunologically active [86,87,88], which fits well with the range of the 33-mer oligomers (10 nm to more than 1 µm). Paul et al. demonstrated that target shape plays a more prominent role than size in the phagocytosis process [89]. The rod-like morphology of gliadin peptides and their capability to form larger superstructures may be sufficient to generate an early immune response and might serve as general disease-causing signals.

As shown previously in macrophages, only the presence of the larger 33-mer structures activates TLR4 and TLR2 [21]. Additionally, the p31-43 oligomers are involved in the inflammasome activation in murine models [26]. Under cumulative conditions due to their inadequate proteolysis, the morphology of the peptide oligomers and particularly the formation of 2D and 3D nano- and microstructures could trigger an early innate response. Unluckily, the presence of canonical T-cell epitopes in the 33-mer sequence triggers the adaptive immune response in CeD patients.

Additionally, it is reported that gliadin acts as a modulator of human microbiota [27], and it can not be discarded that changes in the microbiota due to the presence of gluten participate in GRDs [28,29]. The gliadin superstructures’ presence could also interfere negatively in the initial attachment and subsequent colonization of beneficial bacteria. Another possibility could be that the morphological similarities with pathogenic bacteria favor their attachment and colonization in the mucosa. Notably, both scenarios would lead to dysbiosis, an imbalance of the normal microbiota of the gut [94]. Forsberg et al. showed the first demonstration of morphologically uniform bacteria associated with the mucosa of CeD patients [95], although the presence of bacteria has been implicated in CeD earlier [96,97]. The authors classified bacteria as rod-shaped if the length/diameter ratio was >1.5 and as cocci form if the ratio was ≤1.5 [95]. Bacteria are commonly seen in the intestinal mucosa of patients with active CeD [98]. They are mainly rod-shaped and seemed to adhere to one end between or on the microvilli of the epithelial cells and often appeared in bouquet-like groups [98]. Furthermore, coccoid bacteria were also detected. Moreover, the bacteria either covered the entire surface or were present in large patches covering most of the surface and the lesions observed in CeD patients. Visually, each sample contained bacteria of only one phenotype [95]. Further, in patients with inflammatory bowel disease, a disease sharing several parallels with CeD, attachment of bacteria to the intestinal epithelium was demonstrated [99]. In this regard, dysbiosis is observed in young CeD patients [100]. A recent study has shed light on the interaction between host genetics and dysbiosis with CeD development [101]. Even more, an increase in rod-shaped bacteria in the proximal small intestine microbiota, dominated by *Streptococcus, Staphylococcus,* and *Actinobacteria*, was found to contribute to the development of CeD in young patients [102]. Then, is CeD a risk factor for dysbiosis, or is dysbiosis a risk factor for CeD?

Finally, young CeD patients have unique carbohydrate structures of the glycocalyx/mucous layer, facilitating bacterial adhesion in the proximal small intestine [95]. Although, it was not possible to distinguish between the possibilities that altered glycosylation is an inherited property of individuals who contract CeD or that bacteria influence glycosylation. In this regard, all target proteins show several N-glycosylation motifs (Figure 6 and Figure 7), which reinforce the idea of a potential role as a foreign trigger of CeD. This observation is another example of a relationship between specific pathogens and CeD pathogenesis because pathogen presence could indicate aberrant innate immunity. In these situations, prolamin may irritate the epithelial surface and even be mistaken for a pathogen [95].

Besides the high sequence, structural and morphological similarities reported here, the overall disease course caused by gluten-derived peptides, infection agents, or pathogens share remarkably similar characteristics [6]. First, gluten peptides and pathogens can both enter the intestinal lumen through oral intake. Exposure to most pathogens and gluten is necessary but not sufficient to cause disease because the genetic background and susceptibility, and dose effects, are additional determinants for disease progression [103]. For example, gluten peptides and *Granulicatella* sp. are ubiquitous but only cause disease in susceptible individuals. In this point of view, the flora components alter the homeostasis of the immune system, reshaping the immune environment and promoting the development of specific diseases such as CeD. Therefore, the default setup of intestinal immunity is the generation of tolerance unless specific signals evoke inflammatory reactions. The antigens present in the gut lumen are constantly sampled by intestinal dendritic cells and presented to the T cells in either Peyer’s patches or mesenteric lymph nodes, which results in the generation of regulatory CD4^+^ T cells [104]. Moreover, high concentrations of anti-inflammatory cytokines such as IL-10 and TGF-β are usually found in the intestine of celiac patients [105]. Whereas, speaking about GRDs or bacterial infections, only after their complete elimination from the host body after infection results in remission, and reintroduction causes recurrence of symptoms and/or disease [106].

## 3. Materials and Methods

The α-2-gliadin sequence from *Triticum aestivum* was retrieved from the UniProt database under the accession number Q9M4L6 to obtain the sequence of the 33-mer (57–89 region) and p31-43 (31–43 region) peptides. To identify proteins that share high sequence similarity with the two mentioned relevant celiac peptides, a protein-protein BLAST [30] and an optional SmartBlast search were performed using a BLOSUM62 matrix with gap cost (existence 11 and extension 1). This search was performed using non-redundant protein sequences with a word size of 6 amino acids. Only top-scored hits outside of the *Gramineae* family (taxonomy ID 4479) were used for further analysis.

Protein sequences obtained by BLASTp search, designated as target proteins, were subjected to predicting the natively disordered regions using PrDOS server version 2.0 [107], setting a false positive rate of 5%. After careful manual inspection of those target proteins or related proteins with an experimentally solved structure, respective amino acid sequences were subjected to structural modeling using the protein homology/analogy recognition engine (PHYRE) server version 2.0 [108] in the intensive mode. Only those structure models for which more than 80% of the sequence could be modeled and yielded more than 90% of confidence were selected for further analysis. The models were then subjected to an energy minimization to remove any clashes using a standard protocol in the Amber14 [109] suite as we previously described [19]. The percentage of solvent-accessible surface area (SASA) was calculated using the vmdICE [110] plug-in for VMD 1.9.3 [111] using a water probe radii of 1.4 Å and normalized by the value of the corresponding free amino acid in a GXG peptide context. The structure-based prediction of aggregation hot-spots was performed using AGGRESCAN 3D [112] in the static mode setting a distance cutoff of 10 Å. All protein representations were prepared using VMD 1.9.3 [111]. Subcellular localization prediction was performed with Protter version 1.0 [82].

## 4. Conclusions

Comparing both sets of pathogen-derived proteins presented in Table 1, the p31-43 similarity regions are located mainly in the C-terminal part of the target proteins; meanwhile, the respective 33-mer similarity regions are randomly distributed across the target proteins (Figure 3, Figure 4 and Figure 5). The majority of the similarity regions were identified to be located in disordered or partially disordered regions and solvent-exposed; however, they are located in areas not prone to aggregation in the bacterial proteins (Figure 3, Figure 4 and Figure 5). Interestingly, three out of five of the 33-mer similarity regions are predicted to be located in the extracellular space (Table 1 (A): Hits#3–5, Figure 6). In contrast, the p31-43 similarity regions are predicted to be located intracellular (Figure 7). From the primary structure, gluten peptides and their respective pathogens-related proteins possess PxxP sequences capable of binding predominantly to the SH3 domain and the xPPx (p31-43) motif that binds to WW domains. Therefore, these sequences may potentially bind to a plethora of proteins in vivo, leading to novel metabolic (dis-)functions. Pathological overlaps between the protein and 33-mer peptide, e.g., induction of an immune response, were found for the non-covalent extracellular cbpA from *S. pneumoniae*, which function is to increase the expression of ICAM-1, an early inflammatory maker. In this regard, a specific variant of ICAM-1 with an arginine at position 241 is a predisposing factor for the development of CeD in adulthood. Another significant finding is *Granulicatella* sp., which is found in the gut and reported in the case of CeD, and the corresponding pathogen-related protein has potential celiac disease T-cell recognition motifs. The molecular and structural similarities with *Granulicatella* sp. points out the necessity to investigate the role of these pathogens in the development of CeD by molecular mimicry mechanisms.

Finally, due to similarities between the size and shape of the pathogens to the 33-mer supramolecular structure, we hypothesize that the gliadin peptide capability of forming rod-like and biofilm-similar structures may be connected to the dysbiosis observed in active CeD and the other less understood GRDs. Therefore, it is hypothesized that the 3D patterns formed by the gliadin peptides could play a role in selective bacterial attachment and colonization that can shift the gut homeostasis toward dysbiosis. In addition, the formation of gliadin superstructures could be a pathological trigger that activates the innate immune system, which depending on individual susceptibility, would lead to CeD or the different GRDs. In summary, our findings stress the importance of further experimental research in the field of gut microbiota, particularly with their connection to CeD and other GRDs, with the final aim to improve the health and life quality of gluten-susceptible people.

## Figures and Tables

**Table 1:** Sequence similarity of target protein regions found for the 33-mer (A) and p31-43 (B) peptides of α-2-gliadin.

Protein Name * (Reference)	Organism	Sequence Identity ** (E-Value)	Sequence Similarity (Subcellular and Topological Localization ***)	Organism Pathogenicity in Humans
**BLASTp search using the 33mer sequence as query (A)**				
**Hit#1:** PQQ-repeat protein (NCBI: WP_048584697.1)	*Streptomyces viridochromogenes*	68% (0.004)	^95^PYGQQQDPYGQPQAPYGQPQAPYGQPQP^122^ (T(s-p)/I)	Not reported; producer of bioactive compounds, e.g., antibiotics [31].
**Hit#2:** Aspartate kinase (NCBI: WP_017312730.1)	*Fischerella* sp.	54% (0.064)	^459^PIPNPQSPIPNPQSPIPDPRSPIPDP^484^ (I)	Not reported; producer of secondary metabolites [32].
**Hit#3:** F/YSIRK-type signal peptide-containing protein (NCBI: WP_070445895.1)	*Granulicatella* sp. *HMSC31F03*	46% (0.075)	^1747^QPNPDPEKPTPDPEKPTPDPEKPTPDPE^1774^ (T(s-p)/E)	Potentially pathogenic [33]; commensal of mucosal surfaces.
**Hit#4:** Choline-binding protein A (NCBI: WP_000458116.1)	*Streptococcus pneumoniae* R6	45% (0.079)	^412^KPAEQPQPAPATQPEKPAPKPEKPAEQPK^440^ (E)	Opportunistic pathogen [34]; mucosal surface in upper respiratory tract-forming biofilms.
**Hit#5:** Efflux RND transporter permease subunit (NCBI: WP_015136936.1)	*Nostoc* sp.	54% (0.097)	^535^LPNPQSPIPNPQSPVPNPQSPIPINPL^562^ (T(m-p)/E)	Potentially low pathogenic via ecotoxicology [35]; producer of toxic compounds and bioactive compounds with pharmaceutical potential.
**BLASTp search using the p31-43 sequence as query (B)**				
**Hit#1:** MinD/ParA family protein (NCBI: WP_032757377.1)	*Streptomyces alboviridis*	85% (0.003)	^918^YPGQQQPYPPQQP^930^ (I)	Not reported.
**Hit#2:** MinD/ParA family protein (NCBI: WP_015611830.1)	*Streptomyces fulvissimus*	85% (0.003)	^911^YPGQQQPYPPQQP^923^ (I)	Not reported.
**Hit#3:** MinD-like ATPase (GenBank: SCK04981.1)	*Streptomyces* sp. *ScaeMP-e48*	85% (0.003)	^904^YPGQQQPYPPQQP^916^ (I)	Not reported.
**Hit#4:** Hypothetical calcium-binding protein LENED-006428 (GenBank: GAW04623.1)	*Lentinula edodes*	75% (0.024)	^909^FPSQQQPAPYFPPQP^924^ (I)	Potentially pathogenic [36] causing, e.g., dermatitis herpetiformis; mushroom used in traditional medicine.

* Hits ordered by increasing E-value. ** Sequence identity against 33-mer and p31-43 peptides of α-2-gliadin. *** The sequence and location of the region sharing high sequence similarity with the gliadin peptides within the target proteins are given. Subcellular localization prediction T (s-p or m-p): transmembrane single-pass or multi-pass, E: extracellular, and I: intracellular.

## Data Availability

The data that support the findings of this study are available from the corresponding author upon reasonable request.

## Supplementary Materials

The following are available online at https://www.mdpi.com/article/10.3390/ijms22179278/s1.

## Abbreviations

CeD	celiac disease
ID	intrinsic disorder
MHC	major histocompatibility complex
PPII	type-II polyproline
SASA	solvent-accessible surface area
