# Supplementary material for: Molecular and Structural Parallels between Gluten Pathogenic Peptides and Bacterial-Derived Proteins by Bioinformatics Analysis
Source: Int J Mol Sci. 2021 Aug 27;22(17):9278. doi: 10.3390/ijms22179278 (PMC8430993; doi:10.3390/ijms22179278)
Supplement: Supplementary file 1 [file ijms-22-09278-s001.zip › ijms-1359222-supplementary.pdf]

Supplementary Information

Molecular and Structural Parallels Between Gluten Pathogenic Peptides and Bacterial-derived Proteins by Bioinformatics Analysis

Diego S. Vazquez[1,2,□], Hanna M. Schilbert[3,□,#], and Veronica I. Dodero[3,*]

[1]Grupo de Biología Estructural y Biotecnología (GBEyB-IMBICE), Departamento de Ciencia y Tecnología, Universidad Nacional de Quilmes, Roque Sáenz Peña 352, Bernal, Buenos Aires, Argentina.

[2]Consejo Nacional de Investigaciones Científicas y Técnicas (CONICET), Av. Rivadavia 1917 (C1033AAJ), Ciudad Autónoma de Buenos Aires, Argentina.

[3]Department of Chemistry, Organic Chemistry OCIII, Universität Bielefeld, Universitätsstraße 25, 33615 Bielefeld, Germany.

#Current address: Genetics and Genomics of Plants, Center for Biotechnology (CeBiTec) & Faculty of Biology, Bielefeld University.

□These authors contributed equally to this work.

*Corresponding author: Veronica I. Dodero, E-mail: veronica.dodero@uni-bielefeld.de

Fig S1

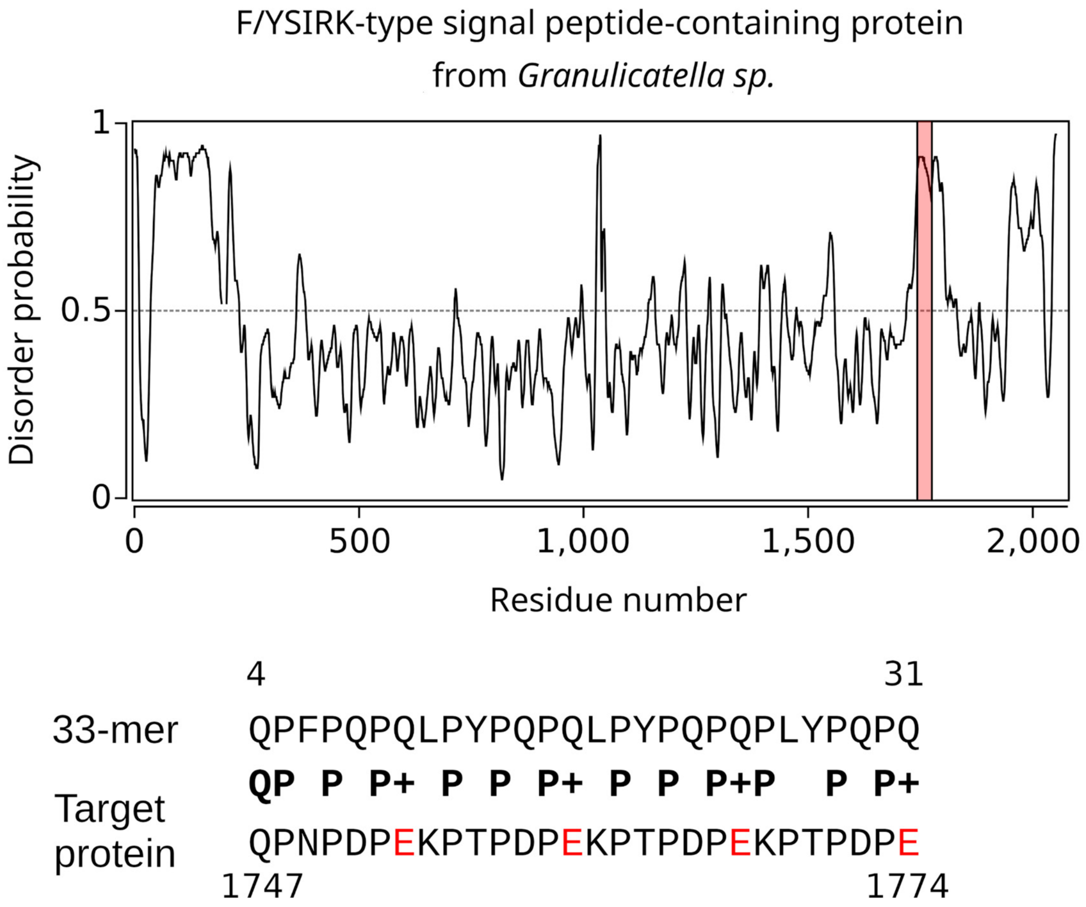

Fig S1. Intrinsic disorder profile of the F/YSIRK-type signal peptide-containing protein from *Granulicatella sp*. HMSC31F03 calculated using PrDOS [1]. The region sharing high sequence similarity with the 33-mer sequence is shown in a red box. In addition, the sequence alignment between the 33-mer sequence and the high similarity region of the target protein is shown at the bottom.

Bibliography

1. Ishida T, Kinoshita K. PrDOS: prediction of disordered protein regions from amino acid sequence. Nucleic Acids Res. 2007;35: W460-4. doi:10.1093/nar/gkm363
